# Supplementary material for: Analysis of behavioral flow resolves latent phenotypes
Source: Nat Methods. 2024 Nov 12;21(12):2376–87. doi: 10.1038/s41592-024-02500-6 (PMC11621029; doi:10.1038/s41592-024-02500-6)
Supplement: Supplementary file 1 — Supplementary Notes 1–3, Tables 1 and 2, and Figs. 1–4. [file 41592_2024_2500_MOESM1_ESM.pdf]

nature methods

Supplementary information https://doi.org/10.1038/s41592-024-02500-6

Analysis of behavioral flow resolves latent phenotypes

In the format provided by the authors and unedited

Supplementary Information

Table of Contents

Supplementary Note 1. Behavioral flow analysis and effect size estimation using different clustering algorithms. **2**

Supplementary Note 2. Applying the analysis pipeline across all experiments with a different clustering approach. **3**

Supplementary Note 3. Methods for behavior testing. **4**

Supplementary Table 1. Manual inspection of clusters pre- and post-transfer **7**

Supplementary Table 2. Feature data **8**

Supplementary Figure 1. .... **10**

Supplementary Figure 2. .... **11**

Supplementary Figure 3. .... **12**

Supplementary Figure 4. .... **13**

References **14**

Supplementary Note 1. Behavioral flow analysis and effect size estimation using different clustering algorithms.

We tested whether the BFA would also work with other clustering algorithms used for analyzing rodent behavior, these are VAME[1] and B-SOiD[2]. For VAME, DLC-based tracking data was used for egocentric alignment as described by the authors (Supplementary Figure 2A) and reached good model performance (Supplementary Figure 2B). Estimating the best number of clusters (as described above) we selected 80 clusters (Supplementary Figure 2C). Similar to the k-means approach, 5 of these clusters revealed significant group differences (adj. $p<0.05$) between CSI and control animals (Supplementary Figure 2D), even though both algorithms identified very different clusters that only correlated moderately (Supplementary Figure 2E). The resulting behavioral flow diagram showed differences for certain transitions (Supplementary Figure 2F), but after multiple testing correction (1820 observed transitions out of 6320 possible transitions), no significant group differences were found (Supplementary Figure 2G). However, BFA - computed based on all behavioral transitions - was able to reveal a highly significant group difference (Supplementary Figure 2H).
We also tested BFA with B-SOiD, which restricted the input features to 9 body points (Supplementary Figure 3A). Adjusting the number of clusters in B-SOiD, which uses a hierarchical clustering method, is not straight-forward, and systematically varying the input parameters generated a wide range of possible clusters. We settled for 8 clusters, which efficiently separated the data (Supplementary Figure 3B), and which were accurately assigned to single frames by the random forest classifier as assessed by confusion matrix and test data accuracy (Supplementary Figure 3C). It has indeed been reported recently that some clustering algorithms only resolve a few behavior motifs in open field data[3], and it offered an opportunity to test whether our BFA approach would work on fewer clusters. Three of the identified clusters showed significant group differences (adj. $p<0.05$; Supplementary Figure 3D). Mapping these B-SOiD clusters to our k-means clusters reveals that every B-SOiD cluster contains many of the clusters represented by k-means (Supplementary Figure 3E). The resulting behavioral flow diagrams show that 5 transitions were significantly different between CSI and controls (adj. $p<0.05$, Supplementary Figure 3F-H), in line with the notion that the small number of observed transitions (56 observed transitions out of 56 possible transitions) rendered the multiple testing correction less punishing. We then conducted BFA and again found that it powerfully resolved the group difference (Supplementary Figure 3I).
Overall, we demonstrate that our analyses based on behavioral flow provide unbiased end-to-end methods for detecting general group differences (BFA) and estimating effect sizes (BFL). These methods demonstrably work with outputs from different established clustering algorithms, and provide a basis for benchmarking available clustering algorithms and selecting optimal hyperparameters.

Supplementary Note 2. Applying the analysis pipeline across all experiments with a different clustering approach.

Thus far, we used k-means to cluster behavioral recordings into behavior motifs. To test if our analysis pipeline (Figure 2C) produces comparable results using a different clustering approach, we re-analyzed all five datasets using VAME for generating 25 clusters. Although the number of significant clusters and transitions differed slightly from the k-means clustering, BFA reproduced significant group differences for all experiments (Supplementary Figure 4A-E), and the approach tended to increase power for detecting group differences compared to the standard OFT readouts. The 2D embedding again showed that all acute stressors (acute swim, yohimbine and DREADD) induced a similar phenotype, while the chronic stressors (CSI and CRS) shifted away from the control groups in the opposite direction (Supplementary Figure 4F). This shows that - even though the VAME clustering did resolve different behavioral motifs compared to k-means clustering (Supplementary Figure 4G) - our analysis pipeline is robust towards the choice of clustering approach and to the represented behavioral motifs.

Supplementary Note 3. Methods for behavior testing.

Open field test (OFT)
Open field testing took place inside sound insulated, ventilated multi conditioning chambers (TSE Systems Ltd, Germany). The open field arena (45 × 45 × 40 cm [L × W × H]) consisted of four transparent Plexiglas walls and a light gray PVC floor. Animals were tested for 10 min under dim lighting (4 lux). Animals were removed from their home cage and placed directly into the center of the open field. The doors of the conditioning chamber were then swiftly closed. Video recording at 25 fps was triggered by infra-red beam break upon the mouse entering the arena.

Marble burying test (MBT)
The marble burying test took place inside the base of a Tecniplast 1500U rat home cage, fitted with a custom 38 x 28 x 45 cm infrared permeable acrylic insert to prevent the mice from escaping. 20 identical, opaque, light green marbles were equally spaced in 4 rows of 5 on top of approximately 2.5-3 cm deep wood chip bedding within the insert. Mice were placed into the center of the arena, after which a camera was triggered to allow video recording at 30 fps. The test was carried out under yellow light between 100-115 lux.

Light-dark box test (LDB)
Light-dark box testing took place inside sound insulated, ventilated multi conditioning chambers (TSE Systems Ltd, Germany). The light-dark box consisted of a two-compartment plexiglass arena and a light gray PVC floor (light compartment: 27.5 x 29.5 x 24.5 cm [L × W × H], 250 lux; dark compartment: 15 x 29.5 x 24.5 cm [L × W × H], 0 lux). Animals could move freely between the two compartments via a cutout in the divider between compartments (7cm x 6.6cm). Animals were removed from their home cage and placed directly into the center of the light compartment. The doors of the conditioning chamber were then swiftly closed. Video recording at 25 fps was triggered by infra-red beam break upon the mouse entering the arena.

Chronic social instability (CSI)
The CSI procedure was carried out as previously described[4] on male C57BL/6J (C57BL/6JRj) mice (n=59) obtained from Janvier (France). The mice arrived at the lab aged between postnatal day 21–23 housed in groups of 5. Upon arrival they were ear-tagged and split randomly (by cage) into either the CSI or control group. The CSI mice underwent the CSI paradigm, which consisted of briefly placing all CSI mice (n = 30) into a larger cage, from which they were then randomly assigned to new cages. Mice in the control group were similarly handled, however, they entered the larger cage only with their cagemates before they were all returned to their original cage. The mice were subjected to these cage changes twice a week (Tuesday/Friday) for seven weeks, during the last cage change the mice were returned to their original cage and allowed to rest for 5 weeks prior to any further testing.

Acute swim (AS)
Male (C57BL/6JRj) mice (n=30) were obtained from Janvier (France). Acute swim (AS) mice (n=15) had to swim in a plastic beaker (20 cm diameter, 25 cm deep) filled to 17 cm with 17.9–18.1 °C water for 6 minutes before they were placed back into their home cages. Control mice (n=15) remained in their homecage until further testing in the open field. Open field testing was performed 45 min and 24 hours after the stress.

Yohimbine injections

For the OFT, male C57BL/6JRj mice (n=20) were obtained from Janvier (France). Before open field testing mice were hand-restrained and received one of 15 dosages of yohimbine ranging from 0.4 mg/kg - 6 mg/kg (n=15) or vehicle (saline 0.9%) (n=5) (i.p.). Mice were immediately placed into the center of the open field test arena following their restraint and i.p. injection.
For the MBT, male C57BL/6JRj mice (n=19) were obtained from Janvier (France). Immediately before testing, mice 3 mg/kg yohimbine (n=10) or vehicle (saline 0.9%) (n=9) (i.p.). Mice were then immediately placed into the center of the marble burying arena and recorded for 30 minutes.

Chronic restraint stress (CRS)

C57BL/6J (C57BL/6JRj) male (n=16) and female (n=16) mice were obtained from Janvier (France) and housed in groups of 4 animals per cage. Upon arrival, animals were randomly split into a control and chronic restraint stress (CRS) group by cage. CRS animals were placed in a 50 ml Falcon tube with a large air hole for 90 minutes per day for 10 consecutive days, while control animals were briefly handled every day. Restraint occurred unpredictably during the dark phase between 10:00 and 17:00. On day 10, CRS animals were placed in the open field test arena 45 minutes after the end of the restraint stress. On day 12, the behavior of all animals was recorded in the light-dark box for 10 min.

Chemogenetic activation of locus coeruleus (DREADD)

Heterozygous C57BL/6-Tg(Dbh-icre)1Gsc female (n=16) mice were subjected to stereotactic brain injections, as described previously[21]. The mice were anesthetized with isoflurane and placed in a stereotaxic frame. For analgesia, animals received a subcutaneous injection of 2 mg/kg Meloxicam and a local anesthetic (Emla cream; 5% lidocaine, 5% prilocaine) before and after surgery. A pneumatic injector (Narishige, IM-11-2) and calibrated microcapillaries (Sigma-Aldrich, P0549) were used to inject 1 µL of virus (ssAAV-5/2-hSyn1-dlox-hM3D(Gq)_mCherry(rev)-dlox-WPRE-hGHp(A); physical titer: 4 x 10E12 vg/ml) bilaterally into the locus coeruleus (coordinates from bregma: anterior/posterior -5.4 mm, medial/lateral ± 1.0 mm, dorsal/ventral -3.8 mm). 3 weeks after surgery, animals were tested in the open field arena for 20 min. For consistency in this study, we only used the first 10 min of recordings. Animals were injected i.p. with 0.03 mg/kg clozapine (Sigma-Aldrich, Steinheim, Germany) (n=8) or vehicle (saline 0.9%, n=8) and placed directly into the center of the open field test arena.

Inescapable footshock (IFS)

Male C57BL/6J (C57BL/6JRj) mice (n=35) were obtained from Janvier (France). Upon arrival, animals were randomly split into a control and inescapable footshock (IFS) group and single-housed one day prior to behavioral testing. Animals stayed single-housed throughout the duration of behavioral testing. All animals were first tested in the open field test for 10 min (OFT1). One day later, IFS animals were placed inside the TSE multi conditioning systems' black fear conditioning arena. After 5 min of rest, the animals received 19 foot shocks (0.5 sec, 1 mA) over 20 min. Shocks were pseudo-randomly distributed over the 20 minutes to occur 30, 60 or 90 seconds apart. Control animals were placed in the same boxes without receiving a footshock. 24 hours later, all animals were placed in the open field test for 10 min (OFT2). All animals were placed in the black fear conditioning arena for 5 minutes daily for 6 consecutive days one day before a final open field test (OFT3).

Yohimbine and Diazepam experiment from Roche

Male C57BL/6J mice (n=64) were obtained from Charles River Laboratories (Saint Germain sur l'Arbresle, France) and single housed in GM500 cages (Tecniplast) upon arrival in the test facility (Roche Innovation Center, Basel) to prevent aggression. Cages were supplemented with nesting material and two pieces of enrichment that were changed during each cage change. Mice were given *ad libitum* access to food (Standard Diet; Kliba Nafag) and water, and temperature and humidity were continuously monitored and controlled to 22°C ± 2°C and 50 ± 10 %, respectively. The holding and test room were maintained on a 12h:12h light: dark cycle, with lights transitioning to fully on by 06:00. Ethical approval for this study was provided by the Federal Food Safety and Veterinary Office of Switzerland. All animal experiments were conducted in strict adherence to the Swiss federal ordinance on animal protection and welfare as well as according to the rules of the Association for Assessment and Accreditation of Laboratory Animal Care International (AAALAC), and with the explicit approval of the local veterinary authorities (License BS2448).

Mice were acclimatized to the facility for 1 week prior to the locomotor activity test. On the day of the test, one cohort of mice (n=8 per treatment group) were randomly allocated to receive either Yohimbine at 1, 3 or 6 mg/kg (i.p.) or its vehicle (0.3% Tween 80 in 0.9% Saline; i.p.), 5 minutes prior to being placed into the center of the test arena. A second cohort of mice (n=8 per treatment group) were randomly allocated to receive either Diazepam at 1, 2 or 3 mg/kg (per os; p.o.) or its vehicle (0.3% Tween 80 in 0.9% Saline; p.o.), 60 minutes prior to being placed into the center of the test arena. The dose volume for both experiments was 10 mL/Kg. Mice weighed on average 24.5g (min / max.: 23g / 27.4g) and 24.0g (min / max.: 20.6g / 26.8g) in the Yohimbine and Diazepam experiments, respectively. The test arena was a clear Perspex chamber (41 x 41 x 30.5 cm), held within a sound- and light-attenuating cubicle (Omnitech Electronics, USA), and retrofitted with a camera positioned above the chamber to enable continuous video capture at 30 fps of locomotor activity during the 30-minute test.

Supplementary Table 1. Manual inspection of clusters pre- and post-transfer

Cluster #	Original Clustering	Transfer Clustering
1	Supported rearing	Supported rearing
2	Movement along the wall	Movement along the wall
3	Center exploration	Center exploration
4	Locomotion in periphery	Locomotion in periphery
5	Clockwise turn	Clockwise turn
6	Exploration/sniffing of the wall	Wall approach and sniffing/exploration
7	Turning away from wall	Turn/move away from wall
8	Slow walk along the wall	Movement along the wall
9	Grooming and unsupported rearing	Grooming and some unsupported rearing
10	Locomotion	Movement in periphery
11	Supported rearing	Supported rearing
12	Slow rearing (supported and unsupported)	Unclear, in corners (very few examples)
13	Movement to/investigation of the corners	Sniffing/exploration of the wall
14	Transition from being stationary to locomotion	Movement towards the center, movement onset
15	Stationary exploration	Unclear (very few examples)
16	Counter clockwise turn	Counter clockwise turn
17	Slow and partial counter clockwise turn in corner	Corner exploration and some supported rearing
18	Unclear, slow exploration (sniffing, walking) some slow rearing	Unclear, sniffing and some slow rearing
19	Fast locomotion along the walls	Locomotion along the wall
20	Small stationary movements (including left/right sniffing)	Stationary with sniffing
21	Approaching the wall	Locomotion from center to periphery
22	Unclear	Unclear locomotion in periphery
23	Unsupported rearing and sniffing (low rearing)	Sniffing + unsupported rearing (few examples)
24	Sniffing, grooming with counter clockwise turn	Slow counter clockwise turn
25	Stop and go, S curve walking	Stop and go, S curve walking (left-right alterations)

Supplementary Table 2. Feature data

Name	Type	Feature scheme
Ac1	acceleration	nose
Ac2	acceleration	headcentre
Ac3	acceleration	neck
Ac4	acceleration	right ear (earr)
Ac5	acceleration	left ear (earl)
Ac6	acceleration	bodycentre
Ac7	acceleration	bodycentre left (bcl)
Ac8	acceleration	bodycentre right (bcr)
Ac9	acceleration	left hip (hipl)
Ac10	acceleration	right hip (hipr)
Ac11	acceleration	tailbase
A1	angle	(hipr - tailbase) - (tailbase - hipl)
A2	angle	(tailbase - bodycentre) - (bodycentre - neck)
A3	angle	(bcr - bodycentre) - (bodycentre -bcl)
A4	angle	(bodycentre - neck) - (neck - headcentre)
A5	angle	(tailbase - bodycentre) - (neck - headcentre)
A6	angle	(bcl - hipl) - (bcl - earl)
A7	angle	(bcr - hipr) - (bcr - earr)
A8	angle	(nose - earr) - (nose - earl)
D1	border proximity	nose
D2	border proximity	neck
D3	border proximity	bodycentre
D4	border proximity	tailbase
S1	distance	nose - headcentre
S2	distance	headcentre - neck

S3	distance	neck - bodycentre
S4	distance	bodycentre - bcr
S5	distance	bodycentre - bcl
S6	distance	bodycentre - tailbase
S7	distance	tailbase - hipr
S8	distance	tailbase - hipl
S9	distance	bcr - hipr
S10	distance	bcl - hipl
S11	distance	bcl - earl
S12	distance	bcr - earr
S13	distance	nose - earr
S14	distance	nose - earl
Ar1	area	tailbase - hipr - hipl
Ar2	area	hipr - hipl - bcl - bcr
Ar3	area	bcr - earr -earl - bcl
Ar4	area	earr - nose - earl

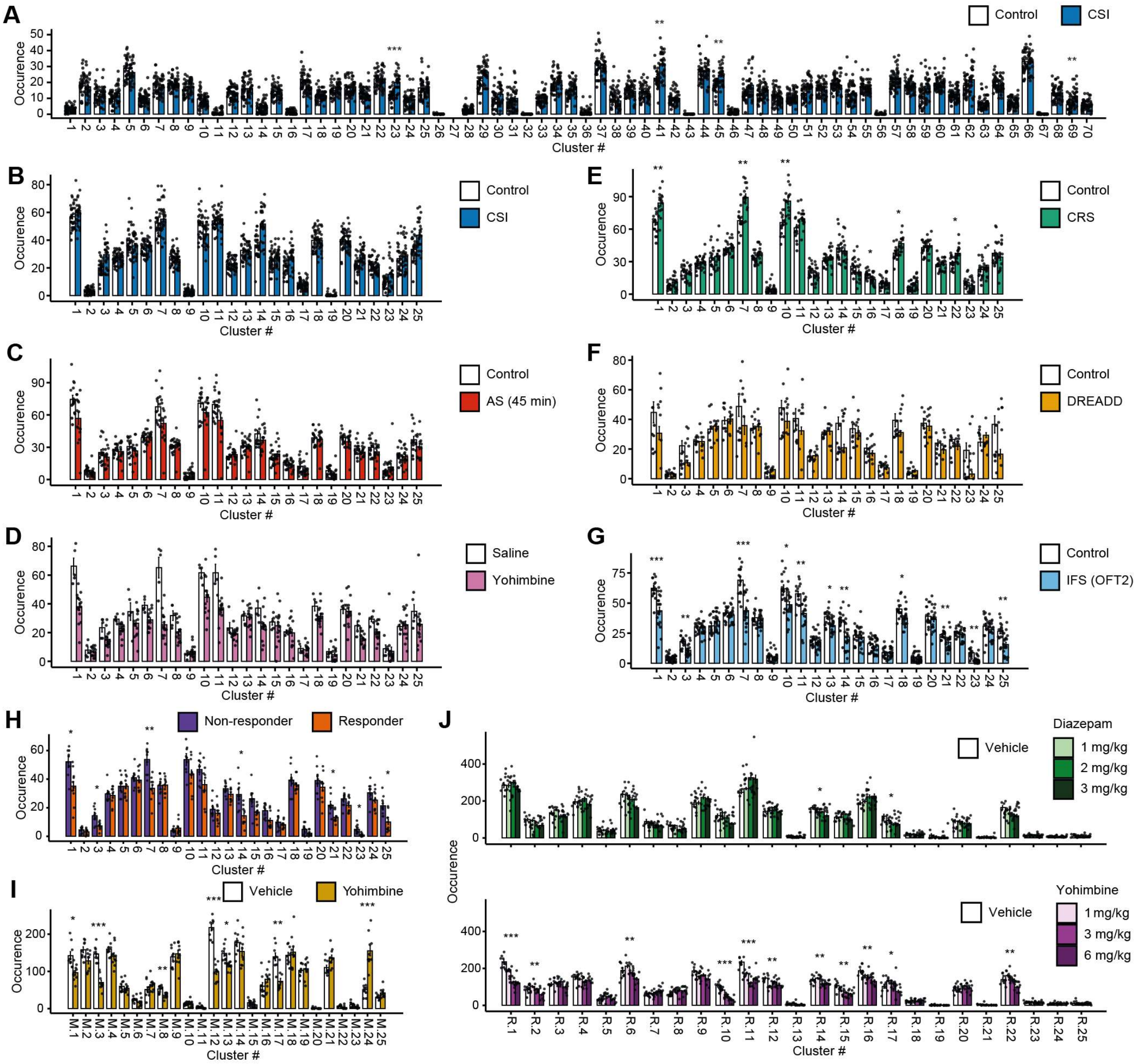

Supplementary Figure 1. k-means cluster occurrences in (A) CSI (n=30, controls: n=29) with 70 clusters, (B) CSI (n=30, controls: n=29) with 25 clusters, (C) acute swim (n=15, controls: n=15), (D) yohimbine (n=15, saline: n=5), (E) CRS (n=16, controls: n=16), (F) DREADD (n=8, controls: n=8), (G) IFS (OFT2: n=20, controls: n=15), (H) non-responding (n=10) vs. responding (n=10) mice after IFS, (I) MBT (yohimbine: n=10, vehicle: n=9; two-tailed t-tests with multiple testing correction), and (J) diazepam (n=24, vehicle: n=8), or in yohimbine (n=24, vehicle: n=8; one-way ANOVA with multiple testing correction). Adj. p-values are denoted as *<0.05, **<0.01, ***<0.001. Error bars in the bar plots denote mean ± SEM.

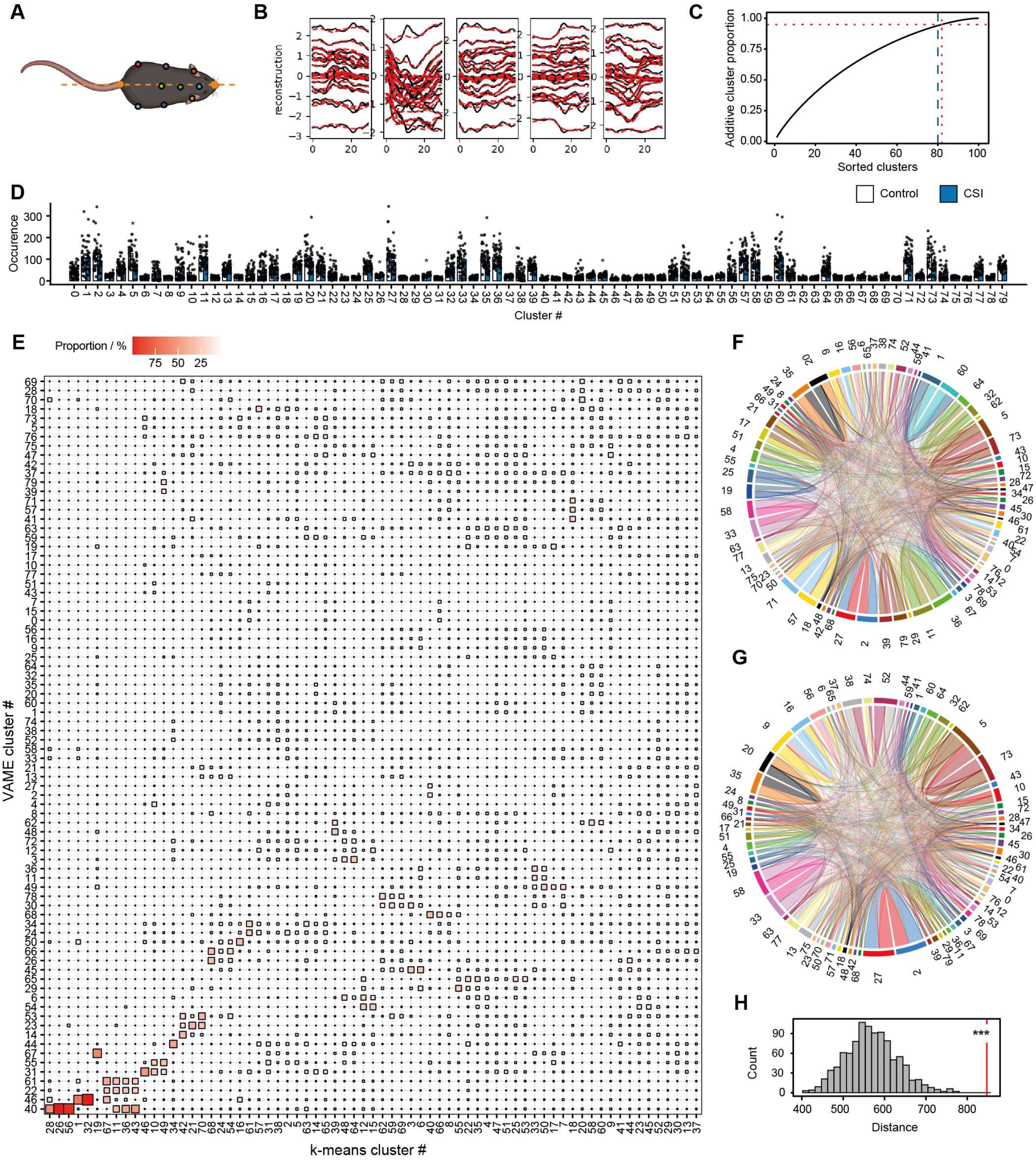

Supplementary Figure 2. (A) Tracking points used for VAME with egocentric alignment (orange dashed line). (B) Reconstruction of tracking points by VAME (over 30 frames). (C) Determining the optimal number of clusters for VAME. Vertical red-dashed line marks the number of clusters (=82) which represent 95% of all frames (horizontal red-dashed line), and the blue dashed line marks the number of clusters (=80) we used for the CSI analysis. (D) VAME cluster occurrences in CSI (n=30, controls: n=29; two-tailed *t*-tests with multiple testing correction). (E) Mapping k-means clusters to VAME clusters. (F) Average behavioral flow over all animals. (G) Absolute difference in behavioral flow in CSI vs. control. For each cluster, the absolute difference in the observed number of transitions between groups is plotted. (H) BFA result for CSI using transitions between VAME clusters (one-tailed *z*-test, percentile=99.9, z=4.42, p=4.97*10^{-6}, d=0.95). p-values and adj. p-values are denoted as *<0.05, **<0.01, ***<0.001. Error bars in the bar plot denote mean ± SEM.

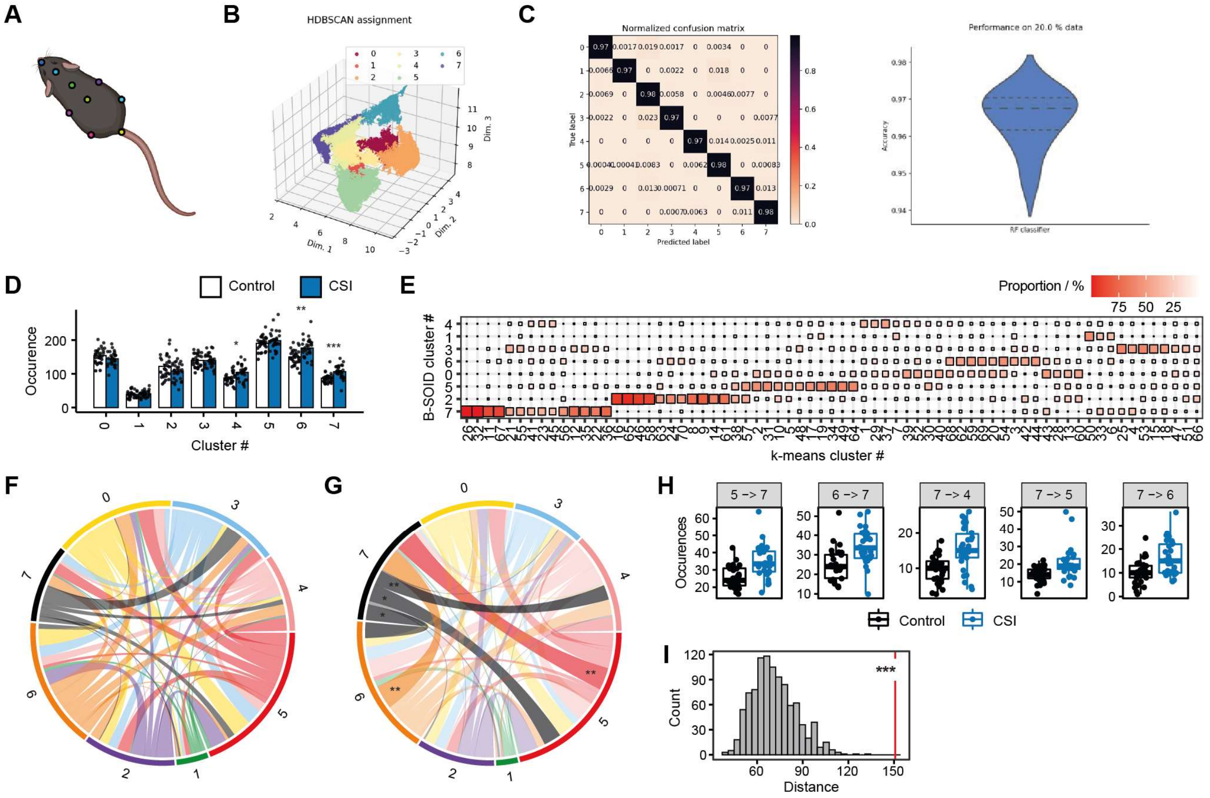

Supplementary Figure 3. (A) Tracking points used for B-SOiD. (B) UMAP visualization of cluster assignment in B-SOiD. (C) Performance of random forest classifier on training (left) and test (right) data. (D) B-SOiD cluster occurrences in CSI (n=30, controls: n=29; two-tailed *t*-tests with multiple testing correction). (E) Mapping k-means clusters to B-SOiD clusters. (F) Average behavioral flow over all animals. (G) Absolute difference in behavioral flow in CSI vs. control. For each cluster, the absolute difference in the observed number of transitions between groups is plotted. (H) Transitions that are significantly different (adj. p<0.05) between CSI and controls. (I) BFA result for CSI using transitions between B-SOiD clusters (one-tailed *z*-test, percentile=99.9, z=5.52, $p=1.69*10^{-8}$, d=1.2). p-values and adj. p-values are denoted as: *<0.05, **<0.01, ***<0.001. Error bars in the bar plot denote mean ± SEM. For box plots, the center line denotes the median value, while the bounding box delineates the 25th to 75th percentiles. Whiskers represent 1.5 times the interquartile range from the lower and upper bounds of the box.

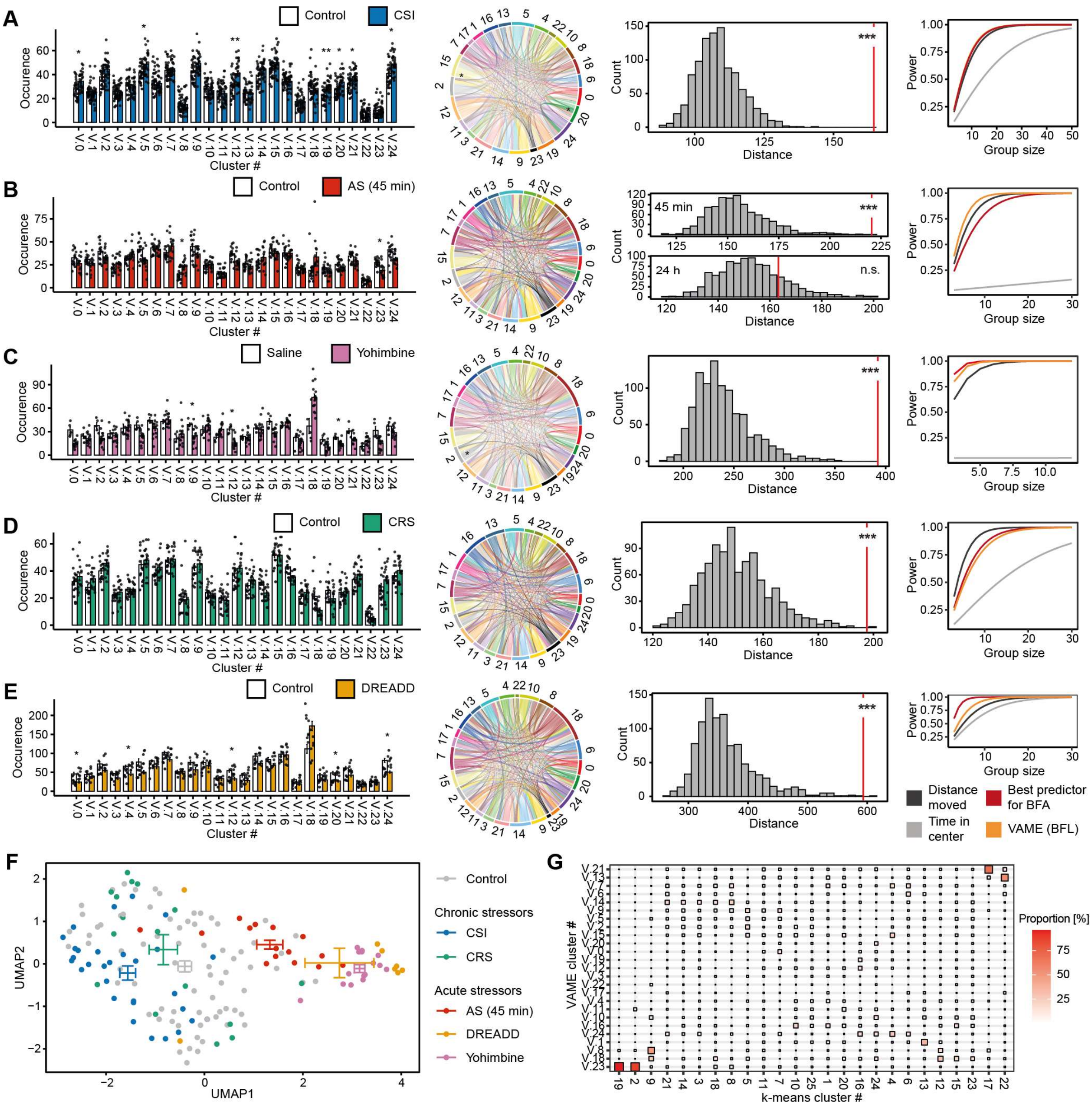

Supplementary Figure 4. Results of our analysis pipeline applied to 25 VAME clusters (V.0-V.24) for (A) CSI (n=30, controls: n=29; one-tailed *z*-test, percentile=99.9, z=6.99, p=1.41*10^{-12}, d=1.26), (B) acute swim (n=15, controls: n=15; one-tailed *z*-test, 45 min: percentile=99.9, z=4.77, p=9.41*10^{-7}, d=1.78; 24 h: percentile=81.42, z=0.83, p=2.03*10^{-1}), (C) yohimbine (n=15, saline: n=5; one-tailed *z*-test, percentile=99.9, z=5.55, p=1.43*10^{-8}, d=3.07), (D) CRS (n=16, controls: n=16; one-tailed *z*-test, percentile=99.8, z=3.87, p=5.53*10^{-5}, d=1.34) and (E) DREADD (n=8, controls: n=8; one-tailed *z*-test, percentile=99.8, z=4.65, p=1.69*10^{-6}, d=1.66). (F) Using the 25 VAME clusters, BFF embeddings across all five experiments (CSI: n=30, AS: n=15, yohimbine: n=15, CRS: n=16, DREADD: n=8, controls (combined): n=73) reveal a separation of different behavioral phenotypes. The crossbars represent the average UMAP1 and UMAP2 values with SEM for each group. (G) Mapping 25 k-means clusters to 25 VAME clusters. p-values and adj. p-values are denoted as *<0.05, **<0.01, ***<0.001. Error bars in the bar plots denote mean ± SEM.

References

1. Luxem, K. *et al.* Open-source tools for behavioral video analysis: Setup, methods, and best practices. *Elife* **12**, (2023).
2. Hsu, A. I. & Yttri, E. A. B-SOiD, an open-source unsupervised algorithm for identification and fast prediction of behaviors. *Nat. Commun.* **12**, 5188 (2021).
3. Bordes, J. *et al.* Automatically annotated motion tracking identifies a distinct social behavioral profile following chronic social defeat stress. *Nat. Commun.* **14**, 4319 (2023).
4. Schmidt, M. V. et al. A novel chronic social stress paradigm in female mice. Horm. Behav. 57, 415–420 (2010).
