# Supplementary material for: Analysis of behavioral flow resolves latent phenotypes
Source: Nat Methods. 2024 Nov 12;21(12):2376–87. doi: 10.1038/s41592-024-02500-6 (PMC11621029; doi:10.1038/s41592-024-02500-6)
Supplement: Supplementary file 2 — Reporting Summary [file 41592_2024_2500_MOESM2_ESM.pdf]

nature portfolio

Corresponding author(s): Johannes Bohacek

Last updated by author(s): Aug 13, 2024

Reporting Summary

Nature Portfolio wishes to improve the reproducibility of the work that we publish. This form provides structure for consistency and transparency in reporting. For further information on Nature Portfolio policies, see our Editorial Policies and the Editorial Policy Checklist.

Statistics

For all statistical analyses, confirm that the following items are present in the figure legend, table legend, main text, or Methods section.

n/a	Confirmed	
☐	☒	The exact sample size (*n*) for each experimental group/condition, given as a discrete number and unit of measurement
☐	☒	A statement on whether measurements were taken from distinct samples or whether the same sample was measured repeatedly
☐	☒	The statistical test(s) used AND whether they are one- or two-sided<br>*Only common tests should be described solely by name; describe more complex techniques in the Methods section.*
☐	☒	A description of all covariates tested
☐	☒	A description of any assumptions or corrections, such as tests of normality and adjustment for multiple comparisons
☐	☒	A full description of the statistical parameters including central tendency (e.g. means) or other basic estimates (e.g. regression coefficient) AND variation (e.g. standard deviation) or associated estimates of uncertainty (e.g. confidence intervals)
☐	☒	For null hypothesis testing, the test statistic (e.g. *F*, *t*, *r*) with confidence intervals, effect sizes, degrees of freedom and *P* value noted<br>*Give P values as exact values whenever suitable.*
☒	☐	For Bayesian analysis, information on the choice of priors and Markov chain Monte Carlo settings
☒	☐	For hierarchical and complex designs, identification of the appropriate level for tests and full reporting of outcomes
☐	☒	Estimates of effect sizes (e.g. Cohen's *d*, Pearson's *r*), indicating how they were calculated

Our web collection on statistics for biologists contains articles on many of the points above.

Software and code

Policy information about availability of computer code

Data collection	Video recording:<br>TSE multi conditioning system (TSE Systems Ltd, Germany)
Data analysis	Pose estimation:<br>Deeplabcut (v2.0.7)<br><br>Unsupervised Clustering:<br>VAME (v1.1) using PyTorch (v1.7.0)<br>B-SOiD (v2.0)<br><br>Analysis:<br>R (v3.6.1)<br>DLCAnalyzer (v1.0)<br>BehaviorFlow (v1.0)<br><br>R packages and version numbers used for analysis:<br>"pwr" v1.3.0<br>"circlize" v0.4.15<br>"imputeTS" v3.2<br>"M3C" v1.16.0<br>"pracma" v2.3.8

"corrplot" v0.92
"keras" v2.8.0
"tensorflow" v2.9.0
"biganalytics" v1.1.21
"reticluate" v1.24

All code for this project has been deposited on github and is freely accessible under https://github.com/ETHZ-INS/BehaviorFlow.

For manuscripts utilizing custom algorithms or software that are central to the research but not yet described in published literature, software must be made available to editors and reviewers. We strongly encourage code deposition in a community repository (e.g. GitHub). See the Nature Portfolio guidelines for submitting code & software for further information.

Data

Policy information about availability of data

All manuscripts must include a data availability statement. This statement should provide the following information, where applicable:

- Accession codes, unique identifiers, or web links for publicly available datasets
- A description of any restrictions on data availability
- For clinical datasets or third party data, please ensure that the statement adheres to our policy

All video data produced in our lab (707 separate recordings), corresponding pose estimation data and metadata has been deposited online and can be accessed under https://zenodo.org/record/8186065 and https://zenodo.org/records/11235068. All video data and pose estimation data produced by Roche (64 separate recordings) can be accessed under https://zenodo.org/record/8188683 and https://zenodo.org/records/11235915.

Human research participants

Policy information about studies involving human research participants and Sex and Gender in Research.

Reporting on sex and gender	N/A
Population characteristics	N/A
Recruitment	N/A
Ethics oversight	N/A

Note that full information on the approval of the study protocol must also be provided in the manuscript.

Field-specific reporting

Please select the one below that is the best fit for your research. If you are not sure, read the appropriate sections before making your selection.

☒ Life sciences ☐ Behavioural & social sciences ☐ Ecological, evolutionary & environmental sciences

For a reference copy of the document with all sections, see nature.com/documents/nr-reporting-summary-flat.pdf

Life sciences study design

All studies must disclose on these points even when the disclosure is negative.

Sample size	Sample size required to detect phenotypes with different analysis methods was determined with an in silico sub-sampling scheme as described in the manuscript. However, datasets were obtained prior to any of these calculations and sample sizes for these datasets were independently determined using power analyses in a number of other projects of the lab.
Data exclusions	No data were excluded for this study.
Replication	For reproducibility assessment we used a number of independently performed experiments that induce acute stress through different means. Additionally, we conducted two independent experiments (diazepam or yohimbine injections) in an external lab (Roche Pharma) to demonstrate transferability of our methods. All attempts of replication were successful.
Randomization	For all experiments we used a block design, where animals from all groups were evenly distributed into multiple smaller blocks, each containing one biological replicate of each group. Processing order was randomized within blocks. During all experimental manipulations the block design was strictly followed to prevent introduction of any uncontrollable, experiment-independent effects.
Blinding	Investigators were blinded during data collection thanks to the randomized block design. Furthermore, the analysis pipeline algorithms (pose estimation, clustering, clustering classifier training, clustering classifier application and calculation of all other metrics such as number of cluster onset/offset/time and transition matrices) are completely blind to group allocation up to the time of statistical testing and annotation on 2D embeddings.

Reporting for specific materials, systems and methods

We require information from authors about some types of materials, experimental systems and methods used in many studies. Here, indicate whether each material, system or method listed is relevant to your study. If you are not sure if a list item applies to your research, read the appropriate section before selecting a response.

Materials & experimental systems

n/a	Involved in the study	
☒	☐	Antibodies
☒	☐	Eukaryotic cell lines
☒	☐	Palaeontology and archaeology
☐	☒	Animals and other organisms
☒	☐	Clinical data
☒	☐	Dual use research of concern

Methods

n/a	Involved in the study	
☒	☐	ChIP-seq
☒	☐	Flow cytometry
☒	☐	MRI-based neuroimaging

Animals and other research organisms

Policy information about studies involving animals; ARRIVE guidelines recommended for reporting animal research, and Sex and Gender in Research

Laboratory animals	C57BL/6J (C57BL/6JRj) mice obtained by Janvier (France) were used for most experiments. Heterozygous C57BL/6-Tg(Dbh-icre)1Gs mice were used for chemogenetic experiments and C57BL/6J mice obtained from Charles River Laboratories (Saint Germain sur l'Arbresle, France) were used for experiments performed at Roche Pharma. Mice from all strains were used for experiments when 2.5–4 months old.
Wild animals	The study did not involve wild animals.
Reporting on sex	A number of experiments were performed with male mice exclusively, one experiment with female mice exclusively and one experiment was balanced with the same number of male and female mice. Metadata in online repositories contain a complete record on sex for each biological replicate. 2D embeddings revealed no apparent sex based effects in experiments performed with both male and female mice. A lot of the experiments contained in this study were performed over several years in our lab, and the lack of sex based designs is due to requirements of other projects in the lab.
Field-collected samples	The study did not involve field-collected samples.
Ethics oversight	All procedures were carried out in accordance to Swiss cantonal regulations for animal experimentation and were approved under licenses: ZH155/2015, ZH161/2017, ZH106/2020, ZH067/2022. Ethical approval for behavior experiments conducted by Roche was provided by the Federal Food Safety and Veterinary Office of Switzerland. All animal experiments were conducted in strict adherence to the Swiss federal ordinance on animal protection and welfare as well as according to the rules of the Association for Assessment and Accreditation of Laboratory Animal Care International (AAALAC), and with the explicit approval of the local veterinary authorities (License BS2448).

Note that full information on the approval of the study protocol must also be provided in the manuscript.
